# Molecular Regulation of Skeletal Muscle Growth and Organelle Biosynthesis: Practical Recommendations for Exercise Training

**DOI:** 10.3390/ijms22052741

**Published:** 2021-03-08

**Authors:** Robert Solsona, Laura Pavlin, Henri Bernardi, Anthony MJ Sanchez

**Affiliations:** 1 Laboratoire Interdisciplinaire Performance Santé Environnement de Montagne (LIPSEM), Faculty of Sports Sciences, University of Perpignan Via Domitia, UR 4640, 7 Avenue Pierre de Coubertin, 66120 Font-Romeu, France; solsonaaa@gmail.com; 2 DMEM, University of Montpellier, INRAE UMR866, 2 Place Pierre Viala, 34060 Montpellier, France; laura.pavlin@inrae.fr (L.P.); henri.bernardi@inrae.fr (H.B.)

**Keywords:** mTOR, eIF3f, protein turnover, ribosome biogenesis, resistance training, endurance training, hypoxia, satellite cells, DNA methylation, epigenetic modifications

## Abstract

The regulation of skeletal muscle mass and organelle homeostasis is dependent on the capacity of cells to produce proteins and to recycle cytosolic portions. In this investigation, the mechanisms involved in skeletal muscle mass regulation—especially those associated with proteosynthesis and with the production of new organelles—are presented. Thus, the critical roles of mammalian/mechanistic target of rapamycin complex 1 (mTORC1) pathway and its regulators are reviewed. In addition, the importance of ribosome biogenesis, satellite cells involvement, myonuclear accretion, and some major epigenetic modifications related to protein synthesis are discussed. Furthermore, several studies conducted on the topic of exercise training have recognized the central role of both endurance and resistance exercise to reorganize sarcomeric proteins and to improve the capacity of cells to build efficient organelles. The molecular mechanisms underlying these adaptations to exercise training are presented throughout this review and practical recommendations for exercise prescription are provided. A better understanding of the aforementioned cellular pathways is essential for both healthy and sick people to avoid inefficient prescriptions and to improve muscle function with emergent strategies (e.g., hypoxic training). Finally, current limitations in the literature and further perspectives, notably on epigenetic mechanisms, are provided to encourage additional investigations on this topic.

## 1. Introduction

Skeletal muscles are essential for the maintenance of body’s integrity and health. Failure in muscle homeostasis leads to physiological integrity impairment, which contributes to chronic pathologies such as cachexia, metabolic and respiratory diseases, chronic inflammation, liver cirrhosis, and sarcopenia [1,2,3,4,5,6]. Muscle deficiency involves detrimental changes including deteriorations of cell metabolism and loss of muscle volume and strength, resulting in poor physical performance and quality of life [7,8,9,10]. Alteration of skeletal muscle mass and strength is in part attributable to dysregulation of the balance between synthesis and breakdown of proteins and other cellular components, such as organelles [11,12,13,14]. Several signal transduction pathways promote muscle atrophy and hypertrophy. Atrophy is the result of increased degradation of cellular proteins and sometimes decreased protein synthesis flux. This state of muscle weakness can be observed in the terminal phase of diseases, such as AIDS, cancer, renal insufficiency, nerve degeneration or metabolic diseases (e.g., diabetes), and even during muscle trauma, immobilization, and aging. Conversely, skeletal muscle hypertrophy is related to enhanced protein synthesis leading to enlargement of pre-existing fibers without variation of myofiber number (i.e., hyperplasia) in humans [11,12,13,14].

The mechanistic/mammalian target of rapamycin complex 1 (mTORC1) was identified as a major regulator of skeletal muscle hypertrophy in response to an increased workload, such as resistance exercise [15,16,17,18,19,20,21,22]. In past decades, mTORC1 was found to regulate a myriad of fundamental muscle physiological processes, including autophagy [23,24,25,26,27,28], cell growth and survival [29,30,31], satellite cells activation associated with muscle regeneration [32,33,34], and ribosome biogenesis [35,36]. Moreover, the mTOR pathway appears important for survival of telomerase-deficient mice displaying short telomeres in the liver, heart, and skeletal muscle [37]. Emerging evidence also suggests that mTORC1 plays a dual role in the development of cancer cachexia [38]. On one hand, reduction of anabolic mTORC1 signaling contributes to loss of muscle mass during cachexia. On the other hand, its inhibition upregulates the autophagy pathway and prevents the production of pro-cachectic factors, protecting from tumor cachexia [38]. Recently, the role of the eukaryotic initiation factor 3f (eIF3f) in mTORC1 activity was highlighted in skeletal muscle [39,40], as well as other regulators, such as diacylglycerol (DAG) and DAG kinases (DGKs) during mechanical stimulation [16,41,42,43,44,45,46,47].

Importantly, the involvement of satellite cells in skeletal muscle mass regulation has been recently questioned but new data highlighted their importance in myonuclear accretion and muscle remodeling during exercise training [48,49,50]. Finally, recent data underlined the importance of an epigenetic regulation (e.g., DNA and histone modifications, expression of specific microRNAs) of skeletal muscle mass, especially during resistance exercise [51,52]. Physical exercise is a common strategy to improve muscle function by enhancing cell size and metabolism [14,53]. Through its remarkable effects on various organs, exercise training has a direct impact on the whole organism by improving global homeostasis (e.g., glucose homeostasis) with a major impact on morbidity [54,55,56]. Chronic exercise affects muscle mass and metabolism through the modulation of fiber composition and size, the improvement of organelle functioning and cell components recycling [14]. Among the multiple mechanisms involved in adaptations to training, increase of muscle mass and strength is associated with enhancement of myofiber cross-sectional area, as well as myofibrillar adjustments, such as transition of myosin heavy chains (MHC) to MHC2A and MHC2X. An improved myosin ATPase activity has also been reported in rats after resistance training [14,28,57]. Importantly, muscle cells translational capacity is critical for muscle mass maintenance and is strongly dependent on ribosome biogenesis [58,59,60,61,62].

This investigation details the mechanisms underlying proteins and organelles biosynthesis in skeletal muscle, especially the mTORC1 pathway, the involvement of eIF3f, ribosome biogenesis, satellite cells, myonuclear accretion, and some major epigenetic modifications. The importance of newly identified regulators of these pathways is discussed, as well as the impact of exercise training and further perspectives to encourage other investigations on this topic. Indeed, numerous studies have recognized the importance of exercise training to improve cell capacity to build proteins and efficient organelles. A deepened knowledge of these mechanisms is critical to provide efficient exercise strategies and to preserve muscle health.

## 2. mTORC1 Signaling, Its Regulators, and Ribosome Biogenesis

The mammalian/mechanistic target of rapamycin (mTOR) is a 289 kDa serine/threonine kinase [63]. mTOR forms two different multiprotein complexes: mTORC1 and mTORC2 [64]. mTORC1 is sensitive to rapamycin and consists of mTOR, regulatory associated protein of mTOR (RAPTOR) [65,66], mTOR-associated protein LST8 homolog (mLST8/GβL) [67], DEP domain containing mTOR-interacting protein (DEPTOR) [68], and proline rich Akt substrate of 40 kDa (PRAS40) [69,70]. mTORC1 was found early in the century to be involved in the regulation of skeletal muscle size and to oppose atrophy [71,72]. On the other side, mTORC2, which is not sensitive to short-duration rapamycin treatment, is composed of mTOR, rapamycin-insensitive companion of mTOR (RICTOR) [73,74], mLST8/GβL [74], DEPTOR [68], and mammalian stress-activated protein kinase interacting protein 1 (mSIN1) [75]. Of note, chronic rapamycin treatment inhibits mTORC2 signaling pathway and promotes insulin resistance via the inhibition of this pathway [76,77]. mTORC2 has been identified as a critical regulator of muscle glucose uptake in response to insulin stimulation and exercise [78]. Importantly, several contractile proteins, actin cytoskeleton regulators, ion-channels, and transcriptional regulators, were suggested as potential substrates of mTORC2 during exercise [78]. mTORC2 is predominantly located at the sarcolemma without modification of this localization in response to feeding and exercise [79].

mTORC1 pathway has been extensively studied in the past few years in skeletal muscle, and particularly during muscle development and growth. In rodent, genetic ablation of mTORC1 components, especially RAPTOR, or pharmacological inhibition of mTORC1, decreases mRNA translation, results in serious muscle dystrophy and prevents overload-induced hypertrophy [71,80,81,82,83,84]. Inhibition of mTORC1 in developing muscle causes perinatal death and negatively affects proliferation and fusion of muscle progenitors during regeneration [85]. Nevertheless, a recent study also highlighted that mTORC1 signaling was not essential for the maintenance of adult muscle size and function five months after complete inhibition of this pathway in sedentary rodents [86]. The authors also found that the expression of critical components of the translation machinery and translation rates were decreased despite stable muscle size and function [86]. Other studies previously showed that effective inhibition of mTORC1 with short-term (one or two weeks) and long-term (four to six months) daily treatments or diet based ingestion of rapamycin does not affect fiber size nor muscle mass [16,87,88,89,90,91,92]. These data suggest that mTORC1 does not play an exclusive role in the maintenance of basal adult skeletal muscle mass but remains important for embryonic and adult myogenesis.

However, mTORC1 plays a critical role in skeletal muscle hypertrophy induced by mechanical stimuli. The study from Baar et Esser in 1999 was the first to suggest a role of mTORC1 pathway in hypertrophy during high-resistance lengthening (or eccentric) contractions [93]. The authors showed for the first time that eccentric actions induced the phosphorylation of the mTOR target p70S6K (70 kDa ribosomal S6 kinase) six hours after exercise, which correlated with the changes of muscle mass observed after six weeks of training [93]. Conversely, submaximal eccentric and maximal concentric contractions do not necessarily impact S6K1 phosphorylation [94]. Then, several studies recognized the role of mTORC1 in exercise-mediated protein turnover and hypertrophy [14]. In addition to these roles, mTORC1 has been recently shown as an essential regulator of the autophagy pathway by inhibiting autophagosome formation through Unc-51-like kinase (ULK1) phosphorylation [23,25,26,27,95]. Interestingly, under mTOR inhibition or amino acid withdrawal, ULK1 phosphorylates Beclin-1 enhancing the activity of the ATG14L-containing Vps34 (vacuolar protein sorting 34) complexe for full autophagy induction [96]. Importantly, sustained activation of mTORC1 in tuberous sclerosis complex 1 (TSC1)-deficient mice blocks autophagy and promotes a late onset myopathy, opening new insides on autophagy-related muscle diseases [97].

**Figure 1:**
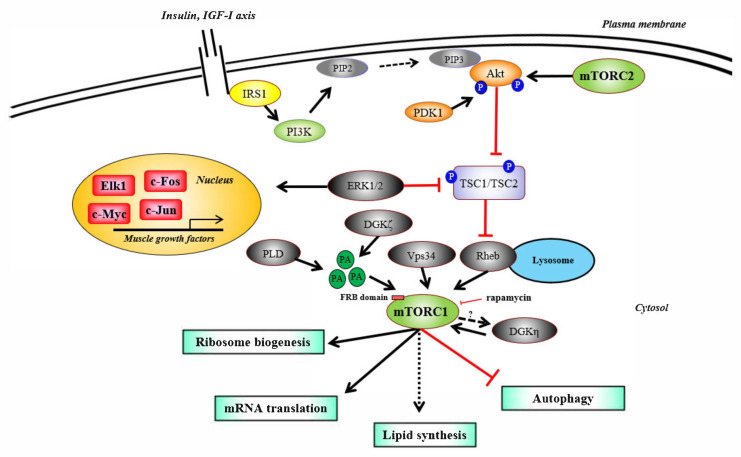
mTORC1 regulates skeletal muscle protein synthesis. Upon IGF-1 (insulin-like growth factor 1) axis activation, IRS1 (insulin receptor substrate 1) activates the lipid kinase PI3K (phosphoinositide 3-kinase). PI3K phosphorylates the membrane-bound phospholipid PIP2 (phosphatidylinositol diphosphate) and generates PIP3 (phosphatidylinositol triphosphate), which recruits Akt/PKB (protein kinase B) and PDK1 (phosphoinositide-dependent kinase-1). PDK1 phosphorylates and activates Akt, which then phosphorylates and inactivates TSC1/TSC2 (tuberous sclerosis complex 1/2), a RHEB (Ras homolog enriched in brain) inhibitor. RHEB then activates mTOR (mechanistic/mammalian target of rapamycin). In response to mechanical stimuli, mTOR is also activated by branched-chain amino acids, Vps34 (vacuolar protein sorting 34), DGKη (diacylglycerol kinase eta) and by PA (phosphatidic acid). PA is synthesized by PLD (phospholipase D) and DGKζ (diacylglycerol kinase zeta) and targets mTOR on its FRB (FKBP-rapamycin-binding) domain to induce its activation. Amino acids promote the recruitment of mTOR to the lysosomal surface, where mTOR is activated by RHEB. ERK1/2 (extracellular signal-regulated kinase 1/2) inhibits TSC1/TSC2 and promotes the transcription of genes involved in muscle growth through Elk1 (ETS-like protein-1), c-Fos, c-Jun and c-Myc. mTOR in association with RICTOR (rapamycin-insensitive companion of mTOR) forms mTORC2 (mechanistic/mammalian target of rapamycin complex 2) and fully activates Akt. mTORC1 (mechanistic/mammalian target of rapamycin complex 1) plays critical roles in mRNA translation, ribosome biogenesis, autophagy inhibition, and perhaps in lipid synthesis in muscle cells.

mTORC1 activity is regulated by the phosphoinositide 3-kinase (PI3K)/Akt (or “protein kinase B”, PKB) axis in response to nutrients and growth factors [98,99,100] (Figure 1). Insulin-like growth factor 1 (IGF-1) receptor is phosphorylated under mitogenic stimuli and recruits the insulin receptor substrate 1 (IRS1), leading to the activation of the phosphoinositide 3-kinase (PI3K). PI3K phosphorylates phosphatidylinositol diphosphate (PIP2) and, thus, generates phosphatidylinositol triphosphate (PIP3), which in turn activates several effectors, including the kinase phosphoinositide-dependent kinase-1 (PDK1). PDK1 phosphorylates and activates Akt, which promotes the inactivation of tuberous sclerosis complex 1/2 (TSC1/2) and the subsequent activation of mTOR through the Ras homolog enriched in brain (RHEB) GTPase [101,102,103,104,105,106,107,108,109,110,111]. Of note, mTORC2 phosphorylates Akt on Ser-473 facilitating Thr-308 phosphorylation by PDK1 [112]. However, muscles expressing a dominant-negative IGF-I receptor show hypertrophy associated with S6K1 phosphorylation in response to mechanical load, suggesting that IGF-1 receptor is not essential for muscle growth [113]. Recently, it was also found that IGF-1/Akt1 regulation is dispensable for activation of mTORC1 signaling and satellite cells following mechanical overload [114]. Furthermore, it was found that mechanical overload induces mTORC1 activation at the early phase thanks to the mitogen-activated protein kinase (MEK)/extracellular signal-regulated kinase (MEK/ERK) pathway through phosphorylation of TSC2 instead of the PI3K/Akt signaling [115]. ERK1/2 pathway is also involved in muscle growth through the regulation of nuclear transcriptional factors (i.e., Elk-1, c-Myc, c-Jun and c-Fos) [116].

Recently, mechanical stimuli were found to activate phospholipase D (PLD) and the lipid second messenger phosphatidic acid (PA) [41]. PA competes with the mTORC1 inhibitor FKBP38 (FK506 binding protein 38), promoting mTOR binding to FKBP12-rapamycin binding (FRB) domain leading to mTORC1 activation [44]. However, none of the IGF-1 and ERK1/2 pathways nor PLD activity modulation appear to be required for mTORC1 activation and skeletal muscle hypertrophy [42,82,114,117]. Nonetheless, recent studies showed that diacylglycerol (DAG) and DAG kinases (DGKs) play an important role in PA accumulation during mechanical stress, especially the zeta isoform of DGK (DGKζ) [45]. In this model, DGKζ stimulates PA biosynthesis through DAG phosphorylation and mTOR activation. PA also binds to the FKBP12-rapamycin binding (FRB) domain of mTOR, promoting the activation of its kinase domain. Moreover, DGKζ represses FOXO3 (forkhead box O3) activity promoting a subsequent inhibition of muscle atrophy F-box (MAFbx)/atrogin-1 and muscle ring-finger protein-1 (MuRF1) expressions [46]. Consistent with these data, DGKζ KO (knock-out) muscles show higher levels of MAFbx/atrogin-1 and MuRF1 after exercise [46]. Finally, it was found that DGKζ is predominantly increased among DGK isoforms during mechanical overload and that DGKζ is essential for muscle growth [46]. Of note, a recent study highlighted that the eta isozyme of DGK (DGKη) promotes myoblast proliferation through the mTORC1/fatty acid synthase (FASN) pathway [47]. The authors also suggest that mTOR and DGKη are mutually regulated since mTOR knockdown reduces DGKη but also FASN expressions [47].

Muscle growth and hypertrophy are not only reliant on the availability of amino-acids and mRNAs, but are also dependent of the translational activity of ribosomes [118]. Ribosomes regulate cellular protein content by assembling amino acids in a sequence indicated by the mRNA to create polypeptide chains. Ribosome biogenesis starts with ribosomal RNA (rRNA) transcription by RNA polymerase I (Pol I) as a 47S pre-rRNA. This long transcript is then cleaved into 28S, 18S, 5.8S and assembled with ribonuclear proteins [119]. Pol I acts like a primary regulator of ribogenesis, forming a pre-initiation complex (PIC) thanks to the transcription initiation factor IA (TIF-1A), the transcription initiation factor IB (TIF-1B), and the upstream binding factor (UBF) [120,121,122]. Of note, transcriptional activity of TIF-1A and UBF mainly depends on cyclins, cyclin-dependent kinases (CDKs), ERK, adenosine monophosphate-activated protein kinase (AMPK), mTORC1, and S6K1 related signaling pathways [120,123,124,125,126,127].

**Figure 2:**
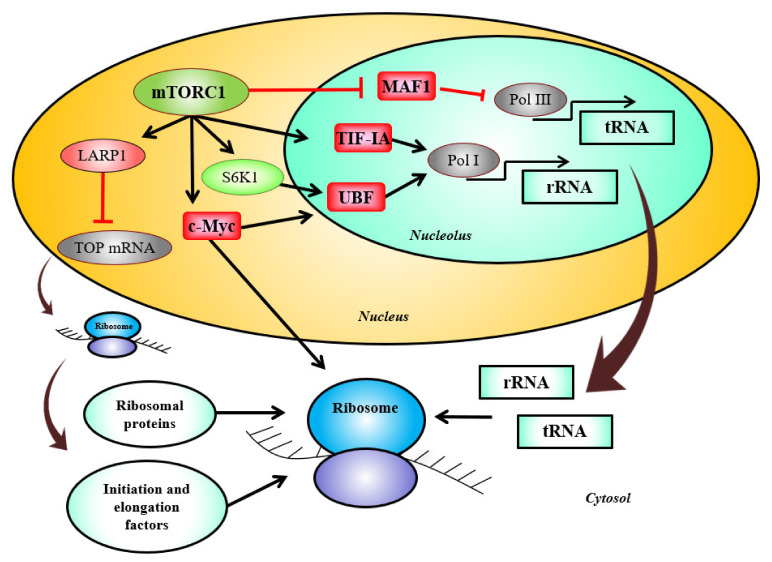
mTORC1 stimulates ribosome biogenesis. mTOR (mechanistic/mammalian target of rapamycin) modulates the activity of TIF-1A(transcription initiation factor IA) involved in pre-ribosomal RNA synthesis and of the ubiquitous transcription factor c-Myc that controls genes products involved in the transcription and processing of rRNA, in the protein components synthesis, and in the nuclear export of ribosomal subunits. mTOR phosphorylates MAF1 leading to inhibition of RNA Pol III (RNA polymerase III) repression function, allowing transfer RNA (tRNA) synthesis. mTORC1 (mechanistic/mammalian target of rapamycin complex 1) interacts with the RNA binding protein LARP1 (La-related protein 1), involved in TOP (terminal oligopyrimidine) mRNAs translation, and subsequently regulates the production of ribosomal proteins and initiation and elongation factors. S6K1 (S6 kinase 1), a master substrate of mTORC1, stimulates RNA Pol I (RNA polymerase I) through activation of UBF (upstream binding factor).

Importantly, mTORC1 signaling pathway regulates ribosome biogenesis at several levels (Figure 2). mTORC1 activates TIF-1A for pre-ribosomal RNA synthesis through Pol I [124]. Moreover, mTOR phosphorylates the human phosphoprotein MAF1 inhibiting RNA polymerase III (Pol III) repression function and leading to subsequent transfer RNA (tRNA) synthesis [128,129]. mTOR also interacts with the RNA binding protein La-related protein 1 (LARP1) involved with terminal oligopyrimidine (TOP) mRNA translation and subsequently regulates the production of ribosomal proteins, as well as initiation and elongation factors [130,131,132]. Finally, mTORC1 regulates the ubiquitous transcription factor c-Myc during chronic resistance training [117]. c-Myc oncoprotein is implicated in the transcription of numerous genes and in the coordination of protein synthesis by upregulating the expression and processing of rRNA and riboproteins components. Transcriptional control of genes required for the initiation of mRNA translation and nuclear export of ribosomal subunits are also modulated by c-Myc [133]. c-Myc is considered as an essential driver of ribosome biogenesis as it regulates the transcription of UBF, TIF-1A, TIF-1B, Pol I, Pol II, and Pol III [133,134,135,136,137,138]. Muscle loading, contractile activity, as well as nutrients availability, hormones, and other growth stimuli may impact ribosome biogenesis through the regulation of the aforementioned pathways [124,139,140].

During hypertrophy, protein synthesis rate increases, muscle protein pool grows but cell translational capacity (ribosomal density) and efficiency (rate of mRNA translation) could be limiting steps. An increase of total RNA and rRNA content was associated with exercise-induced hypertrophy [58,141,142,143]. In accordance with these observations, the precursor 45S and mature rRNA 18S, 28S and 5.8S transcripts levels were elevated during a resistance training protocol in humans [142]. rRNA content and ribosomal biogenesis are determinant for hypertrophy in human skeletal muscle after a resistance training regimen, and blunted ribosomal production in vitro impedes myotubes hypertrophy [58]. The increase in total RNA and rRNA content seems to be correlated with the muscle growth response [58], suggesting that ribosome biogenesis is a key factor for generating higher hypertrophy in “super-responder” subjects. In mice, early increases in rRNA and 45S-pre-rRNA content, as well as expression of c-Myc and its downstream Pol I regulon, were found following functional hypertrophy [144]. Interestingly, chromatin remodeling at the ribosomal DNA (rDNA) promoter was also observed in the same study [144], showing that both transcriptional and epigenetic mechanisms are involved in ribosome biogenesis at the onset of muscle hypertrophy. Finally, West and coworkers recently found that both rapamycin-dependent and rapamycin-independent pathways (e.g., myostatin) are involved in ribosome biogenesis in response to resistance training [145].

In summary, mTORC1 signaling pathway is essential for embryonic and adult myogenesis, but not for adult skeletal muscle mass maintenance under normal conditions. mTORC1 stimulates ribosome biogenesis at numerous levels and is critical for hypertrophy in response to mechanical stimuli, even if research on mTORC1-independent mechanisms is still limited. Importantly, among DGK isoforms, DGKζ is now considered as a key regulator of protein biosynthesis during overload. In addition to its role on mTORC1 regulation, DGKζ also prevents protein breakdown by inhibiting FOXO3 pathway. Additional studies are needed to better understand the precise role of DGKζ on catabolic systems (i.e., proteasomal and autophagy activities) and studies in humans could be desirable from a translational perspective.

## 3. eIF3f and mTORC1 in Skeletal Muscle Function

**Figure 3:**
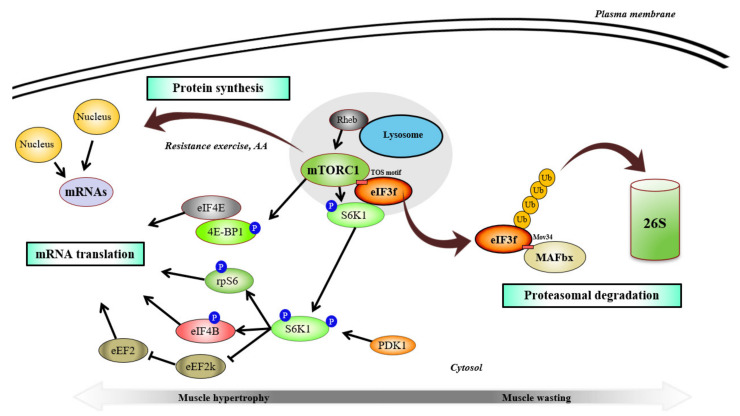
mTORC1 regulates eIF3f in skeletal muscle protein translation. RHEB (Ras homolog enriched in brain) activates mTORC1 (mechanistic/mammalian target of rapamycin complex 1), which binds to the scaffold protein eIF3f (eukaryotic initiation factor 3f ) through a TOS (TOR signaling) motif and modulates mRNA translation by phosphorylating eIF3f bound-S6K1 (S6 kinase 1) and 4E-BP1 (eukaryotic translation initiation factor 4E-binding protein 1). S6K1-mediated regulation of translation occurs, in part, through phosphorylation of rpS6 (ribosomal protein S6), eIF4B (eukaryotic translation initiation factor 4B), and eEF2k (eukaryotic elongation factor-2 kinase). eEF2k inhibits eEF2 (eukaryotic elongation factor-2) to promotes mRNA translation. Phosphorylation of 4E-BP1 by mTOR (mechanistic/mammalian target of rapamycin) promotes its dissociation from eIF4E (eukaryotic initiation factor 4E) and allows for the assembly of the preinitiation complex. During muscle wasting, eIF3f is targeted by MAFbx (muscle atrophy F-box) at Mov34 motif to promote eIF3f proteasomal degradation. Resistance training and amino acids enable the migration of the mTORC1/eIF3f complex at the lysosome surface and at the cell membrane near to peripheral nuclei and blood capillaries. Of note, PDK1 (phosphoinositide-dependent kinase-1) phosphorylates S6K1 for full activation.

The initiation of eukaryotic mRNA translation needs the cooperation of 12 eukaryotic initiation factors called “eIFs” forming numerous complexes. These complexes promote mRNA attachment to the ribosomal 40S subunit, mRNA scanning, start codon selection, and accommodation of initiator tRNA at the P site of the 40S subunit [146]. The mRNA eukaryotic translation initiation factor complex eIF3 is a large complex of about 800 kDa composed of 13 subunits that is essential for protein synthesis [147,148]. eIF3 has a role at different steps of mRNA translation, including (i) the assembly of the ternary complex eIF2-GTP-Met-tRNAi and its recruitment to the 40S ribosomal subunit to form the preinitiation complex 43S PIC and (ii) the mRNA recruitment to the 43S PIC and scanning for AUG codon recognition [146,149,150]. In mammals, an important regulator of mTORC1 activity and muscle hypertrophy is one of these subunits, the initiation factor eIF3f [40,151] (Figure 3).

eIF3f is a member of the Mov34 family with a conserved Mpr1/Pad1/N-terminal (MPN) domain. eIF3f possesses a TOS (TOR signaling) motif (FETML, amino acids 323–327) allowing eIF3f to serve as a scaffold protein to connect mTORC1 with its translational target S6K1 [151,152]. Thus, upon mitogen/growth factor/amino acid stimulation, mTOR phosphorylates its downstream effectors to initiate mRNA translation to proteins [153]. When activated, S6K1 releases from eIF3f and phosphorylates several substrates involved in translation, such as the ribosomal protein S6, the eukaryotic elongation factor 2 (eEF2) kinase (eEF2k), and the eukaryotic translation initiation factor 4B (eIF4B) [151,154,155]. Of note, PDK1 catalyzes S6K1 activity through phosphorylation on residue Thr-229 for full activation [156]. In addition to its role in mRNA translation, S6K1 was also shown to regulate the transcriptional program of ribosome biogenesis [157]. S6K1-dependent regulation of this transcriptional program includes the control of nucleolar protein 56 (Nop56), nucleolar protein 14 (Nop14), GAR1 ribonucleoprotein (“GAR” for glycine/arginine-rich), rRNA processing 9 (Rrp9), rRNA processing 15 (Rrp15), rRNA processing 12 (Rrp12), and periodic tryptophan protein 2 (Pwp2) nucleolar proteins [157]. S6K1 also controls Pol I through the phosphorylation of UBF enhancing rRNA synthesis (Figure 2) [158]. Furthermore, mTOR-mediated phosphorylation of 4E-BP1 (eukaryotic translation initiation factor 4E-binding protein 1) promotes its dissociation from the eukaryotic translation initiation factor 4E (eIF4E) favoring the assembly of the PIC and the recruitment of the eukaryotic translation initiation factor 4G (eIF4G) at the 5’ end of mRNAs [159]. Thus, eIF3f acts as a scaffold protein to support the initiation of cap-dependent translation in mammals.

However, eIF3f possesses an ambivalent function according to the cell type. Indeed, eIF3f negatively regulates cell growth by inhibiting both cap-dependent and cap-independent translation and by increasing rRNA degradation in cancer cells [160,161]. In this model, rRNA degradation is carried out by direct interaction between eIF3f and heterogeneous nuclear ribonucleoprotein (hnRNP) K, a RNA-binding protein required for maintaining rRNA stability [161]. eIF3f interaction with hnRNP promotes hnRNP dissociation from rRNA leading to rRNA degradation [161]. eIF3f also reduces tumor growth by interacting with the secretory heterodimeric glycoprotein clusterin and interrupting its anti-apoptotic property [162]. Interestingly, eIF3f interacts with the N-terminal region of the spike protein of severe acute respiratory syndrome coronavirus (SARS-CoV) and avian coronavirus infectious bronchitis virus (IBV) [163], opening windows for possible research on treatment of coronavirus-diseases (COVIDs). As cardiac abnormality and musculoskeletal dysfunction have been reported in COVID-19 [164,165], this research area is currently of interest.

In recent years, the physiological role of eIF3f has been more extensively investigated in skeletal muscle. It was found that the E3 ubiquitin ligase MAFbx/atrogin-1 targets the Mov34 motif of eIF3f during atrophy for polyubiquitination and subsequent degradation by the proteasome [40,166,167]. A series of studies highlighted that eIF3f expression is essential to maintain skeletal muscle mass and that expression of a mutant (eIF3f K5-10R, where six lysine residues on the C-terminal have been mutated) insensitive to MAFbx/atrogin-1 targeting protects against starvation-induced muscle atrophy [39,40,151,167]. eIF3f K5-10R overexpression also promotes enhanced protein synthesis and hypertrophy in normal conditions compared to wild type protein, suggesting a role of eIF3f in muscle growth [40,151]. Of note, a subset of microRNAs (miRNAs) in the delta-like homolog 1 and the type III iodothyronine deiodinase (Dlk1-Dio3) cluster showed an anti-atrophic effect, probably by limiting the degradation of eIF3f by MAFbx/atrogin-1 [168].

Importantly, it was found that mice carrying a null mutation of the *eIF3f* gene failed to develop and died at the early embryonic stage [39]. One candidate explaining the development impairment could be the Notch pathway known to be positively regulated through eIF3f deubiquitinase activity [169]. Because Notch is essential for post-implantation development [170], a possible defect of this pathway in eIF3f null mice could contribute to failed development. However, heterozygous mice (eIF3f^+/-^ mice) live but show a lower skeletal muscle mass associated with a lower mTORC1 pathway activation, polysome content and protein synthesis flux [39]. Noteworthy, muscle atrophy (i.e., muscle mass and CSA) was found to be exacerbated in heterozygous mice compared to control during immobilization [39]. Of note, no impact of partial deletion of eIF3f was found on both the ubiquitination levels of muscle proteins and numerous markers of autophagy pathway, including markers of mitophagy (i.e., the specific degradation of mitochondria through autophagy) [39]. Thus, eIF3f plays a critical role in embryonic development and adult skeletal muscle mass maintenance, with a role essentially focused on protein synthesis pathways rather than proteolysis.

Furthermore, a role of eIF3f in adaptations to exercise was recently suggested. Mechanical overload enhances eIF3f expression and, interestingly, this effect is blunted in DGKζ KO muscles [46]. Thus, inhibition of FOXO3 pathway by DGKζ could play a role in MAFbx/atrogin-1-induced eIF3f degradation and mRNA translation. Importantly, studies with immunofluorescence approaches found a new cellular trafficking involving mTORC1/eIF3f during exercise [171]. In humans, mTOR was found to co-localize with the lysosomal-associated membrane protein (LAMP2) at rest, suggesting that mTOR is located close to the late-lysosome in skeletal muscle [172]. A single bout of resistance exercise increased the translocation of mTOR/LAMP2 complex at the cell membrane near to the blood capillaries [172]. However, it was previously indicated that mTORC1 recruitment to the lysosome membranes is essential to rise mTOR kinase activity, especially because the mTORC1 activator RHEB is contained in a membrane-bound compartment of the lysosome [16,173,174,175]. Importantly, the authors also found that the interaction between mTOR and eIF3f increased at the cell membrane after exercise and this response was enhanced in a fed state (20g protein/ 40 g carbohydrate/ 1 g fat) [172]. That probably explains the increase of S6K1 kinase activity and protein synthesis enhancement observed after exercise with amino acids ingestion [172,176,177]. Accordingly, mTORC1 translocation to lysosomes is known to be mediated by Ragulator-Rag complex and plays a pivotal role in amino acid signaling related to mTORC1 [174]. Of note, a reduction of TSC2 abundance at the cell membrane was also observed after exercise with a dissociation from RHEB [172]. These results strongly suggest that this newly identified trafficking contributes to higher activation of mTOR signaling.

Interestingly, recent data reinforced our knowledge on the influence of nutritional state after exercise on mTOR/eIF3f trafficking and adaptations to resistance training. Indeed, it was reported that the ingestion of whole eggs after resistance exercise promotes a higher stimulation of protein synthesis compared to consumption of egg whites only [178]. A more pronounced mTOR localization at the lysosome was observed after whole eggs consumption [179]. Among the multiple candidates involved in mTOR trafficking after whole egg ingestion, PA and low-density lipoprotein (LDL)-derived cholesterol have been suggested [178]. The first one can be generated via de novo synthesis by phosphatidylcholine, oleic acid, and diacylglycerol [180,181]. Egg yolks also contain LDL-derived cholesterol known to have a role in mTORC1 recruitment to the lysosome through SLC38A9-Niemann-Pick C1 signaling complex [182]. Altogether, these data suggest that whole egg ingestion could promote a higher mRNA translation compared to egg white. However, even if these acute outcomes may suggest that whole eggs consumption could maximize gains in skeletal muscle mass, longitudinal data recently failed to support this assumption [183].

A recent study compared the effect of daily whole egg vs. egg white ingestion during 12 weeks of resistance training in humans [183]. The authors found that, when protein intake was equalized, the effects of whole egg and egg white on body weight, fat mass, skeletal muscle mass, and strength, and the expression of fibroblast growth factor 2, follistatin, transforming growth factor-beta1, activin 1, and myostatin were similar. Thus, optimizing acute protein synthesis with nutritional strategies does not necessarily support long-term adaptations in skeletal muscle. Finally, another recent study assessed the effects of a habitual high-protein diet on response to an acute resistance exercise and muscle protein synthesis [184]. The authors found that a four-week high-protein diet (crude protein ≈ 52%) does not influence resting mTORC1 activity nor muscle protein synthesis, as well as in response to a single bout of resistance exercise. However, higher muscle mass and lower fat mass were found with habitual high-protein diet compared to control, but with a concomitant impairment in glucose metabolism-related proteins [184]. Consistent with this result, long-term habitual high-protein intake may promote insulin resistance and whole body glucose intolerance [185].

In summary, knowledge has been significantly improved on eIF3f/mTORC1 axis and the biological significance of eIF3f in the past few years. Thus, eIF3f appears as a critical regulator of mTORC1 axis and is essential for embryonic development and skeletal muscle mass maintenance. In addition, the lysosome is suggested as a key regulatory site of eIF3f/mTORC1 axis. Further investigations have to be encouraged on metabolism and in the context of diseases, aging, and physical activity to better understand the potential interest of this axis as a therapeutic target. Finally, caution may be warranted for prediction of long-term outcomes and investigations have to be encouraged to examine the effects of long-term nutritional strategies, including protein-diet approaches, on whole body homeostasis.

## 4. Implication of Satellite Cells and Myonuclear Accretion

**Figure 4:**
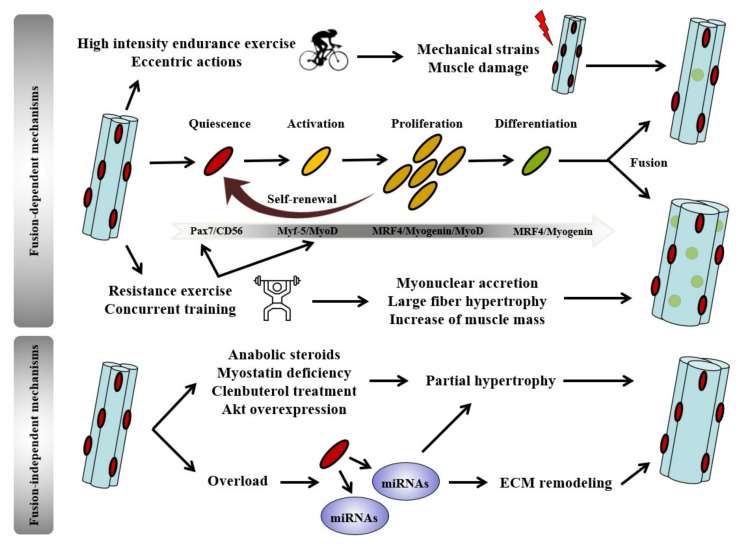
Fusion-dependent and independent mechanisms of satellite cells during growth stimuli. Adult satellite cells (SCs) are in a quiescent state, expressing Pax7 (paired box protein 7) and CD56 (cluster of differentiation 56). Activation of SCs sets off the progression of myogenic lineage from quiescence to fusion. It requires MRF (myogenic regulatory factors) such as Myf-5 (myogenic factor 5), MyoD (myoblast determination protein D), myogenin, and MRF4 (myogenic regulatory factor 4). Whereas damaging exercises (high intensity endurance and eccentric contractions) promote SCs fusion in a regeneration process, exercise-induced hypertrophy requires SCs fusion and myonuclear accretion to increase fiber size. However, fusion-independent mechanisms may promote partial hypertrophy. The secretory functions of SCs seem to play a role in these mechanisms and in ECM (extracellular matrix) remodeling through miRNA-containing exosomes release.

One remarkable property of muscle tissue is to continuously renew itself thanks to the presence of muscle stem cells, also referred as satellite cells (SCs) discovered in 1961 by Alexander Mauro [186]. SCs are mononucleated cells located between the basal lamina and the plasmalemma of myofibers. Upon stimulation, SCs have the ability to drive out of their quiescent state to modulate their gene expression profile, and to start proliferating. These activated SCs termed myoblasts can stop their proliferation and differentiate into muscle progenitors (myocytes), which fuse with existing myofibers. Fusion of myoblasts with preexistent myofibers can lead to enhance myonuclear number (referred to *“myonuclear accretion”*) [187,188]. Adult SCs are in a quiescent state, expressing both paired box protein 7 (Pax7) and cluster of differentiation 56 (CD56) myogenic factors, and get activated under muscle stimulation or injury [189,190]. SCs myogenic lineage progression requires myogenic regulatory factors (MRFs), such as myogenic factor 5 (Myf-5), myoblast determination protein D (MyoD), myogenin, and myogenic regulatory factor 4 (MRF4). Activation of quiescent SCs occurs with the simultaneous increased expression of Myf-5 and MyoD. A subset of activated SCs downregulates MyoD expression to self-renew the quiescent SCs pool. Another subset undergoes myogenic commitment by subsequent expression of MRF4 and myogenin whereas Pax7, CD56, and Myf5 factors are no longer expressed. Differentiation then occurs and myoblasts fuse with preexisting myofibers while MyoD expression decreases [191] (Figure 4). Whereas the role of SCs in fiber damage repair and remodeling is well described, SCs implication in muscle growth and hypertrophy has been debated for a long time in adult skeletal muscle.

The addition of new myonuclei takes place during myofiber hypertrophy thanks to SCs, providing additional cytoplasmic volume in fiber syncytium [192,193,194]. On the contrary, muscle inactivity decreases SCs proliferation [195] and muscle atrophy is associated with a loss of myonuclei [196,197]. Activation of SCs occurs within skeletal muscle following a single bout of exercise [198] and, conversely, abrogation of muscle progenitors seems to impede exercise-induced hypertrophy [199,200,201]. Exercise is known to be an essential anabolic stimulus for muscle tissue, and it was shown that, according to the type of intervention, exercise modulates SCs activation for fiber repair or hypertrophy. Studies on endurance training reported conflicting results on the involvement of SCs, probably due to differences in exercise intensity, volume and duration, as well as the muscle type analyzed [191,202]. An increase in SCs activation was reported after exhaustive eccentric endurance exercises in mice [203]. Accordingly, it was suggested that new myonuclear accretion induced by continued muscle loading may be associated with fiber repair and regeneration after exercise-induced injury [204]. In humans, SCs activation seems to be more related to exercise intensity and eccentric actions rather than duration of endurance exercises, these training modalities promoting higher mechanical strains and muscle damages [198,205,206,207]. Sustained and intense endurance exercise can induce the activation of SCs to ensure muscle damage repair and it remains difficult to attribute SCs activation to the hypertrophic response rather than to a regeneration process [208,209].

Long-term endurance or resistance training was found to increase the pool of SCs in both rodents and humans, as well as combination of endurance and resistance training (i.e., “concurrent training”) [207,210,211]. Of note, resistance exercise is more prone to induce muscle hypertrophy than endurance exercise, and results are less conflicting concerning SCs response. Burd et al. reported an elevation of Pax7 expression following a non-damaging low-intensity resistance exercise but expression of other markers of SCs activation (MyoD and myogenin) was upregulated only when a higher volume of the same exercise was achieved [212]. Studies using resistance exercises established that one bout of moderate to high intensity upregulates SCs activation with an increase in expression of myogenic factors, such as CD56, Pax7l, and MyoD [191]. The effect of resistance exercise on SCs activation is supported by numerous evidences and converge towards an increase in both SCs content and activation, as well as myonuclear accretion, a few hours after and until 4 days post-intervention [191]. Supporting that SCs implication is dependent on stimulus amplitude, exercise was recently found to induce SCs fusion in a load-dependent manner whatever muscle typology [213]. However, in response to resistance training, the number of SCs increased mainly in type II myofibers whereas type I myofibers were almost not affected [211,214,215]. This is consistent with the fact that type II fibers have a greater contribution to hypertrophy and a better adaptive potential than type I. Noteworthy, while resistance training increases SCs pool, myonuclear accretion and muscle mass, endurance training does not necessarily promote myonuclear accretion or increase of muscle mass [209,216,217]. Endurance training appears more effective to increase satellite cell pool when exercise intensity is high, exercise duration being less susceptible to influence satellite cell content [209].

Besides exercise, numerous experimental conditions, such as steroids administration, nutritional interventions, or overloading models promote hypertrophy and conversely may attenuate atrophy in a disease setting. A great accrual of myonuclei was previously observed during a pronounced hypertrophic response following testosterone treatment in mice [218]. However, when a moderate muscle hypertrophy was induced by the same treatment, neither satellite cells number nor myonuclear accretion were found to enhance muscle fiber size [219]. Here, again, intensity of the stimulus seems to influence SCs activation and subsequent terminal differentiation and fusion with myofibers. Furthermore, hypertrophic responses induced by various stimuli, from pharmacological to genetic interventions, were not always associated with increased myonuclear number. This was the case when hypertrophy was induced by genetic ablation of myostatin [220], treatment with the beta 2-adrenergic agonist clenbuterol [221] and overexpression of the serine/threonine kinase Akt [222] (Figure 4). Of note, lysine supplementation, known to stimulate muscle growth, is associated with mTORC1 pathway activation and SCs proliferation/differentiation in piglets [223,224].

In the past decade, McCarthy’s group used Pax7-DTA-mediated ablation of SCs and suggested that muscle hypertrophic response to overloading could be independent of satellite cells intervention [225]. However, these results were recently debated by Egner and coworkers who found a large overload-induced hypertrophy, which was prevented in satellite cell-deficient mice under the same experimental conditions [201]. In accordance with these findings, other methodological approaches based on SCs inhibition by ɣ-irradiation indicated that muscle hypertrophy was prevented when SCs recruitment was blunted [199]. The conditional depletion of more than 90% of SCs of transgenic Pax7-DTA mice yielded also blunted hypertrophic and myonuclear accretion responses [226]. By using young mice genetically deleted of fusion-required serum response factor, Randrianarison-Huetz confirmed the essential role of SCs for overload-induced hypertrophy [227]. In the same way, CDK1 deletion impaired SCs proliferation and showed strong limitation of overload-induced hypertrophy [228].

In a study from Goh et al., authors abrogated fusion of muscle progenitors by specifically deleting myomaker (i.e., a membrane protein involved in myoblast fusion). Results revealed that myonuclear accrual and hypertrophic response were blunted following synergistic ablation [229]. Importantly, another study from Goh and coworkers addressed the question if myonuclei accrual is required in a physiological setting that did not use supra-physiological models. Authors submitted mice to high-intensity interval training (HIIT) during eight weeks and demonstrated that abrogation of muscle progenitor cells fusion at the onset of the protocol blocked myonuclear accretion and hypertrophic response [187]. In addition, muscle fibrosis was observed, as well as exercise intolerance in myomaker-deficient mice. To distinguish differential requirement of muscle progenitor cells fusion between early and late stages, the authors also abrogated muscle progenitor cells fusion after four weeks of training. Results showed attenuated hypertrophy although mice no longer presented exercise intolerance or fibrosis. Authors suggested that myonuclear accretion promotes muscle repair in the early stage of the training protocol and muscle growth during the last stage. Finally, maturational ages are of importance in the involvement of SCs and myonuclear accretion during postnatal growth. Indeed, it was suggested that in response to overload, muscle fiber hypertrophy was prevented in young SCs depleted mice, but not in adult mice (>four months old) [230]. It was also shown that SCs deletion reduced myonuclear number, prepubertal myofiber hypertrophic growth, and force generation [231]. According to these findings, there is evidence that SCs fusion plays an active role in post-natal muscle hypertrophy that can be dependent on factors as age.

However, as hypertrophy was not totally blunted when SCs were impaired in some studies [226,232], fusion-independent mechanisms may also have a role within the muscle tissue to ensure partial hypertrophy. The canonical function of SCs during hypertrophic response of adult myofibers is myonuclear accretion via cells fusion. Nevertheless, Murach et al. underlined that SCs also display fusion-independent roles through their secretory functions, showing that SCs communicate with muscle fibers without necessarily achieve fusion [233]. They previously demonstrated that SCs communicated with fibrogenic cells through exosomes to ensure correct regulation of the extracellular environment in response to hypertrophic stimuli [234]. This fusion-independent mechanism probably involves miRNAs that affect extracellular matrix [234]. Using a mice model of delayed SCs fusion, authors provided evidence that SCs also released extracellular vesicles to muscle fibers [233]. From their results, authors gave novel insights about SCs functions during hypertrophy. Thus, they suggest that coordination of the early hypertrophic response to overload may also be dependent of fusion-independent mechanisms (Figure 4).

During short-term anabolic steroid treatment, the extra-nuclei acquired were not lost months after treatment arrest whereas muscle size had returned to its basal value [218]. From these results, emerged the “muscle memory” theory that differentiates at least two different hypertrophic responses: (i) when adult muscle has no hypertrophic response history, recruitment of new myonuclei is required for de novo muscle hypertrophy, and (ii) when mature muscle has already been submitted to an anabolic stimuli leading to hypertrophy (e.g., resistance training) with a previous increase in the myonuclear number, the addition of new myonuclei seems not needed during a second exposure inducing regrowth [201]. Several studies demonstrated that muscle growth was not necessarily accompanied by an increase in myonuclear number, and, supporting the “muscle memory” theory, a pool of extra nuclei was already contained in muscle fibers during regrowth of muscle tissue. Studies giving evidence in favor of this hypothesis included various models of unloading and reloading where myonuclear number remained unchanged [200,201,232,235].

So far, studies failed to provide conclusive results on human skeletal muscle tissue (for review see [236]). Human training and detraining data indicate that myonuclear accrual found during muscle hypertrophy is reverted during detraining with a loss of myonuclei [237]. As suggested by authors [237], the muscle memory of hypertrophic response may be independent of myonuclear number, and probably due to myonuclear DNA methylation, histone modifications, miRNA expression, and other epigenetic mechanisms. As underlined in the study of Murach et al. [48], miR-1 downregulation after a resistance training program remains lower after six months of detraining and could contribute to make a kind of memory induced by a first training adaptation to facilitate regrowth during further exposure. Reinforcing the role of miRNAs in skeletal muscle mass maintenance, it was suggested that miR-21 expression in SCs and muscle could inhibit myogenesis in old mice and contribute to the decline in muscle regeneration during aging [238]. Other miRNAs candidates, such as miR-23a, miR-27a, miR-29b, miR-29c, and others warrant further attention since they are involved in skeletal muscle mass regulation [239,240,241,242]. Finally, chronic resistance exercise also showed modifications in methylation levels of a myriad of genes. Filamin B (FLNB), myosin heavy chain 9 (MYH9), SLIT-ROBO Rho GTPase activating protein 1 (SRGAP1), serglycin (SRGN), Zinc Finger MIZ-type containing 1 (ZMIZ1) genes were found to be hypomethylated after acute and chronic exercise, and remained hypomethylated during detraining [52].

Taken together, results showed that SCs activation and myonuclear accretion have an important role during uninjured adult muscle growing and contribute to muscle hypertrophy. Results obtained in mice indicate that factors, such as the type of growth stimulus, its magnitude, and the age of animals directly influence SCs involvement. However, data are still lacking in humans. Concerning exercise modality, resistance training, or high-intensity and damaging endurance exercise seems to be the most effective conditions to solicit SCs. Finally, recent data indicate that epigenetic mechanisms could be involved to facilitate future growth in response to resistance training after a period of detraining. Altogether, these data suggest that the role of SCs, myonuclear accretion, and epigenetics modifications still represent an important challenge for further research in post-developmental muscle growth.

## 5. Impact of Exercise Training and Practical Recommendations

Skeletal muscle mass trends to be attenuated during numerous states, such as disuse atrophy or during aging, and it is well recognized that exercise training minimizes cellular disturbances during such states. Acute exercise decreases protein synthesis according to exercise duration and intensity while mTORC1 pathway is reactivated during recovery [21,243]. For example, 45 min of running with a progressive increase in velocity for the last 20 min has been shown to decrease puromycin incorporation in several muscles [244], indicating a global decrease of protein synthesis flux during exercise. Interestingly, 10 sessions of resistance exercise interspaced by only 48 h of recovery blunts ERK/MAPK signaling and mTORC1 activation over time [245]. This probably contributes to the attenuation of protein synthesis during repeated bouts of resistance exercise with short recovery [245,246]. Chronic exercise promotes cellular adaptations leading to improvement of mitochondrial function, reducing oxidative damage and attenuating the rate of skeletal muscle mass decline. A recent study examined the effects of disuse muscle atrophy on mTORC1 activation and muscle protein synthesis during a single bout of resistance training, and whether disuse muscle atrophy could interfere with muscle mass and strength gains after a resistance training protocol [247]. In this work, a 14-day hindlimb suspension decreased basal rRNA level, but not mTORC1 activity and muscle protein synthesis in rats. Importantly, the response of muscle hypertrophy did not differ between the groups [247], showing that disuse muscle atrophy does not alter muscle protein synthesis in response to acute resistance exercise and muscle hypertrophy in response to chronic resistance training. Consistent with these data, another study on disuse atrophy showed that intermittent loading with protein ingestion prevents atrophy during hindlimb unloading probably through mTORC1 signaling pathway [248].

Several factors may impact adaptations to training, such as training backgrounds, nutritional strategies, recovery, exercise modality, exercise volume, and intensity, rest between exercise bouts, genetic/epigenetic factors, age, and environmental conditions [14,59,61,243,249]. First of all, it is important to note that concurrent exercise (i.e., the incorporation of resistance and endurance training into an exercise program) in untrained individuals promotes generic molecular responses leading to whole muscle adaptations (i.e., increases of aerobic and strength aptitudes) and higher gains in muscle mass [250,251,252]. However, while endurance-trained subjects present an activation of S6K1 in response to resistance exercise, no effect can be detected in strength-trained individuals [253]. On the contrary, during endurance training, the metabolic sensor AMPK, which is a major inhibitor of mTORC1 pathway [24], can be increased in strength-trained athletes [253]. An investigation in mice showed that whereas a single bout of exercise substantially increases S6K1 and rpS6 phosphorylation, the chronicity of exercise results in a significant attenuation of this response [254]. Interestingly, S6K1 and rpS6 phosphorylation levels were restored after a short detraining period (i.e., 12 days) [254]. Thus, anabolic response seems less sensitive to resistance exercise with chronic exercise and a detraining period may restore mTORC1 pathway response.

Another study investigated the involvement of epigenetics mechanisms in response to acute resistance exercise in sedentary and trained men. The authors found that resistance-trained subjects showed hypermethylation of the metabolic genes sterol regulatory element-binding protein (SREBF2) and glycerol-3-phosphate acyltransferase, mitochondrial (GPMA). A lower methylation level of SREBF2 was observed in sedentary subjects [255]. This result supports that generic molecular responses and global muscle adaptations occur in sedentary subjects. However, more specific adjustments take place when subjects become adapted. In this study, it was also found that resistance training does not affect methylation of mTOR and Akt [255], which is not surprising since training rather promotes post-translational modifications (i.e., phosphorylation) on these proteins [14,40,243]. Altogether, these results suggest that the training state is an important modulator of molecular signaling pathways, including those involved in protein synthesis. Interestingly, it was recently found that exercise modality during resistance training may finely influence the nature of adaptations. For example, short-term high-volume resistance training increases fiber cross sectional area but reduces fiber actin and myosin protein content in trained young men [256]. A sarcoplasmic expansion can be observed concomitantly with an upregulation of sarcoplasmic proteins involved in glycolysis and other metabolic processes linked to ATP generation [256]. These effects seem to persist up to 8 days following training [256]. Thus, these data show that short-term high-volume training may induce gains in skeletal muscle mass through a sarcoplasmic hypertrophy.

Furthermore, it is important to consider that there are individual variations in training responses with athletes experiencing positive adaptations and some subjects exhibiting no clear improvement of performance and even adverse responses [257]. It was suggested that differences in individual responses may be due to technical measurement errors, limited number of measured variables, differences in individual history and genotype, and other factors (i.e., psycho-emotional states, sleep hygiene, nutritional intake, etc.) [257]. An important factor implicated in the mitigated response to resistance training is ribosome content [59,61,62,258]. For example, blunted ribosome biogenesis was observed in the elderly with a lower hypertrophic response [59,259,260,261]. Recent studies have compared the influence of low- and high- intensity resistance training during aging. No meaningful differences were observed in hypertrophy, improvement of muscle strength, and quality of life [262,263,264].

Of note, training intensity has to be manipulated with caution because frailty frequently occurs in aged people that can be subjected to traumatic injuries. Importantly, it was suggested that low-load high volume, but not high-intensity resistance training, promotes gains in endurance during aging, even if both conditions have similar positive effects on peak oxygen consumption [265]. Some negative mitochondrial adaptations have also been reported with maximal strength training in old subjects [266]. However, while exercise intensity does not appear to be a key factor in adaptations to resistance training in the elderly, manipulating training volume appears most relevant. With a contralateral protocol, a study highlighted a dose-dependent relationship between muscle adaptations and training volume [62,258]. Moderate training volume induced more pronounced gains in muscle strength and mass, as well as a greater ribosome biogenesis and type II fiber transition compared to low training volume [62,258]. A previous study underlined that resistance exercise conducted at low-load and high-volume was more effective than high-load and low-volume to stimulate myofibrillar protein synthesis in young men [212]. In addition, as mitochondrial dysfunctions also occur during muscle disuse or aging, resistance training at low intensity but high-volume can be recommended as it may improve physical functioning [267,268,269,270,271,272,273,274]. It was demonstrated that resistance training may promote ameliorations in walking endurance, muscle oxidative capacity and strength [275,276,277]. In aged people, the benefits of resistance exercise on muscle function were recently attributed to improvement of mitochondrial function, muscle hypertrophy, modulation of myonuclear domain, and newly formed myonuclei, increases of satellite cell-capillary interaction and content [49,278,279]. Of note, it appears that Nordic walking also improves lower limb strength, aerobic aptitude, body composition, life, and sleep quality in old people [280].

Finally, eccentric actions and hypoxic stress have emerged as promising strategies over the last few years. Eccentric actions may promote more robust mTORC1 pathway activation and increases of skeletal muscle mass and strength compared to concentric and isometric contractions [281,282,283]. Of note, normalization of the force-signal integral to a same magnitude effect leads to similar acute molecular anabolic responses [284,285,286,287]. A recent study showed that combined maximal concentric and eccentric training does not produce greater gains in muscular isometric strength and hypertrophy than maximal concentric training alone in young males [288]. Low-intensity eccentric actions should be used in frail people, such as aged subjects, to minimize the occurrence of muscle damage and the risk of a traumatic injury. Furthermore, combination of resistance exercise with hypoxic stress promotes several advantages. Indeed, in the last decade, investigations have been conducted on supplementation of hypoxia during resistance training. Reduction of intramuscular oxygen partial pressure promotes greater cellular stress in skeletal muscle and may improve several aspects of performance, including aerobic capacity and sprint ability in athletes [289,290,291].

However, in older adults, hypoxic resistance training does not induce greater magnitude in gains of lean mass and muscle strength than normoxic resistance training [292]. Moreover, hypoxic training promotes similar changes in oxidative metabolism and insulin sensitivity than exercise in normoxia in older individuals [293]. However, cognitive performance may be improved with intermittent hypoxic training in aged people [294]. That said, the combination of eccentric endurance exercises with low hypoxia may present some advantages on muscle function during aging [295]. Importantly, among hypoxic methods, blood flow restriction (BFR, also called “occlusion training”) is increasingly considered. BFR consists in the generation of local hypoxia in skeletal muscles or restricted venous return during exercise depending on the occlusive pressure level [296]. BFR promotes additional effects on both aerobic and resistance training adaptations. During resistance training, BFR can promote improvements in muscle size, strength, and athletic performance [296]. Resistance training with BFR also promotes proliferation of myogenic stem cells, myonuclei addition, as well as enhancement of angiogenic genes expression [297,298]. Recently, it was found that BFR can limit skeletal muscle atrophy and MuRF1 expression during cast immobilization and muscular weakness induced by chronic unloading [299,300]. In addition, there are some data about BFR during muscle wasting or aging and a case report has suggested that BFR should be considered to improve physical fitness, prevent muscle loss, and improve arterial compliance in frail aged subjects [301,302]. In old women, resistance training at low intensity combined with BFR at 110 mmHg appears effective to induce hypertrophy and gains in muscle strength [303], and walking with BFR at higher pressure improves limb venous compliance [304]. However, a study reported that a six-week walking training with BFR did not improve peak oxygen uptake in old men and women, even if functional ability, muscle size, and strength were improved [305]. Finally, in postmenopausal women with osteopenia or osteoporosis, low intensity resistance training with BFR may also be an effective method to induce bone formation markers [306].

Thus, resistance training promotes several advantages during muscle disease including effects on skeletal muscle mass, strength, and metabolism. Among the factors involved in training adaptations, training volume increase appears critical to benefit from resistance training effects, especially in aged people. More recently highlighted, changes in ribosome biogenesis are more pronounced with multiple set training than with single-set regimen. Chronic resistance exercise has the potential to stimulate both protein translation and ribosomal biogenesis in a volume-dependent manner. In aged people, there is evidence that local hypoxic training (i.e., BFR) is beneficial to promote additional adaptations than normal training. Supplementary studies using BFR (e.g., gravity induced-BFR [307]) have to be encouraged since it represents an easy method that promotes effects on both aerobic and strength adaptations. In another way, further research on nutritional interventions, such as BCAAs/leucine supplementation efficiency in combination with resistance training, especially in frail population and giving concrete guidelines, would be relevant in a perspective of personalized medicine.

## 6. Conclusions and Perspectives

**Figure 5:**
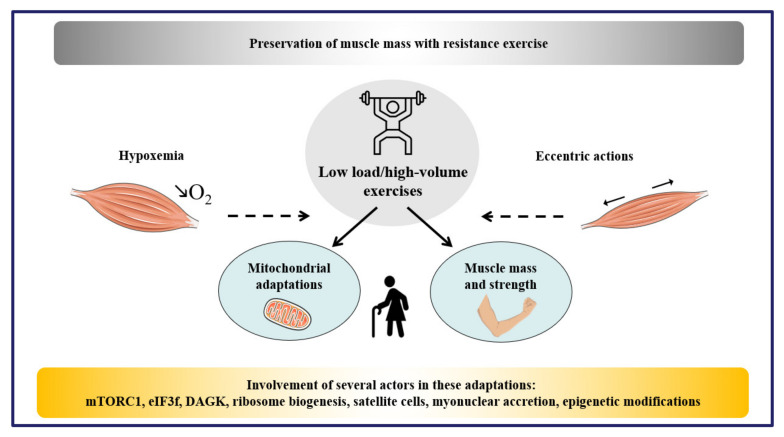
Resistance exercise and muscle mass preservation. Resistance training promotes hypertrophy and low-intensity/high-volume training may induce both improvements in muscle mass and strength, as well as mitochondrial adaptations in aged people. Further studies have to be encouraged to better understand the impact of resistance training combined with hypoxic stress and eccentric actions during muscle atrophy. mTORC1, mechanistic/mammalian target of rapamycin complex 1; eiF3f, eukaryotic initiation factor 3f; DAGK, diacylglycerol kinase.

The regulation of skeletal muscle protein synthesis is a key event for skeletal muscle growth and hypertrophy, especially in response to exercise training (Figure 5). The development of strategies to struggle against atrophy is fundamental to improve the quality of life or the capacity of sick people to recover from illness. Thus, it is imperative to better understand the cellular and molecular mechanisms involved in skeletal muscle failure, as well as the impact of chronic exercise and additional nutritional strategies on muscle dysfunctions. mTORC1 controls embryonic and adult myogenesis without being essential for adult skeletal muscle mass maintenance under normal conditions. However, mTORC1 is essential for muscle growth and hypertrophy under mechanical stimulation, especially by controlling mRNA translation and ribosome biogenesis at multiple levels. After resistance exercise, translocation of mTOR to the lysosome surface and to the cell membrane leads to mTOR association with its regulators, RHEB and eIF3f, and appears consistent with a raised mRNA translation. Importantly, physical exercise is a powerful modulator of the aforementioned pathway, which needs to be more investigated in order to identify the best approaches to counteract atrophy in the long-term. The impact of resistance training has garnered increasing attention, notably in the context of aging. Resistance training appears beneficial in the elderly, especially when training with high-volume and low intensity is privileged. Of note, investigations on ribosomal turnover, including ribophagy (i.e., the degradation of ribosomes through autophagy) are still limited and need further attention.

## Data Availability

Not applicable.
